# Assessment of Calcium Score Cutoff Point for Clinically Significant Aortic Stenosis on Lung Cancer Screening Program Low-Dose Computed Tomography—A Cross-Sectional Analysis

**DOI:** 10.3390/diagnostics13020246

**Published:** 2023-01-09

**Authors:** Kaja Klein-Awerjanow, Witold Rzyman, Robert Dziedzic, Jadwiga Fijalkowska, Piotr Spychalski, Edyta Szurowska, Marcin Fijalkowski

**Affiliations:** 1 Second Department of Radiology, Medical University of Gdansk, 80-210 Gdansk, Poland; 2 Department of Thoracic Surgery, Medical University of Gdansk, 80-210 Gdansk, Poland; 3 Department of General, Endocrine and Transplant Surgery, Medical University of Gdansk, 80-210 Gdansk, Poland; 4 First Department of Cardiology, Medical University of Gdansk, 80-210 Gdansk, Poland

**Keywords:** low-dose computed tomography, LDCT, lung cancer screening, screening, aortic valve stenosis, AS, aortic valve calcification

## Abstract

Low-dose computed tomography (LDCT) is predominantly applied in lung cancer screening programs. Tobacco smoking is the main risk factor for developing lung cancer but is also common for cardiovascular diseases, including aortic stenosis (AS). Consequently, an increased prevalence of cardiovascular diseases is expected in lung cancer screenees. Therefore, initial aortic valve calcification evaluation should be additionally performed on LDCT. The aim of this study was to estimate a calcium score (CS) cutoff point for clinically significant AS diagnosis based on LDCT, confirmed by echocardiographic examination. The study included 6631 heavy smokers who participated in a lung cancer screening program (MOLTEST BIS). LDCTs were performed on all individuals and were additionally assessed for aortic valve calcification with the use of CS according to the Agatston method. Patients with CS ≥ 900 were referred for echocardiography to confirm the diagnosis of AS and to evaluate its severity. Of 6631 individuals, 54 met the inclusion criteria and underwent echocardiography for confirmation and assessment of AS. Based on that data, receiver operating characteristic (ROC) curves of CS were plotted, and cutoff points for clinically significant AS diagnosis were established: A CS of 1758 for at least moderate AS had 85.71% (CI 65.36–95.02%) sensitivity and 75.76% (CI 58.98–87.17%) specificity; a CS of 2665 for severe AS had 87.5% (CI 73.89–94.54%) sensitivity and 76.92% (CI 49.74–91.82%) specificity. This is the first study to assess possible CS cutoff points for diagnosing clinically significant AS detected by LDCT in lung cancer screening participants. LDCT with CS assessment could enable early detection of patients with clinically significant AS and therefore identify patients who require appropriate treatment.

## 1. Introduction

The use of computed tomography (CT) in aortic valve assessment has developed significantly in recent years. Clevel et al. proved that the degree of valve calcification seen in multidetector CT is related to aortic stenosis (AS) severity. The measurements obtained in their study, which are currently expressed as calcium score (CS), have provided prognostic value for survival, as an additional test, along with clinical and echocardiographic (ECHO) assessment [1,2]. Subsequently, the CS results assessed on CT were included in the European Society of Cardiology (ESC)/European Association for Cardio-Thoracic Surgery (EACTS) guidelines as a criterion that increases the likelihood of detection of severe AS [3].

Recent studies proved that the initial aortic valve calcification evaluation can also be performed on low-dose CT (LDCT), which is mainly applied in lung cancer screening programs. These programs are offered to at-risk populations—former and current smokers [4,5,6,7,8]. Notably, this risk factor is common for both lung and cardiovascular diseases, including AS [9]. Consequently, an increased prevalence of cardiovascular diseases can be expected in lung cancer screenees.

AS is the most common valvular disease in developed countries [10]. The only available treatment method is valve replacement [3,11]. It can be performed either surgically or, in selected cases, through a catheter. Therefore, the aim of this study was to estimate a CS cutoff point for clinically significant AS, confirmed by ECHO examination. This cutoff point could enable identification of patients who require specific treatment.

## 2. Material and Methods

This study was a cross-sectional analysis of data from a cohort of 6631 participants of the MOLTEST BIS study, which was a LDCT lung cancer screening research project conducted in Gdansk between 2016 and 2018 in an at-risk population defined as individuals aged 50–79 with a smoking history of ≥30 packs year [5]. The study was approved by the independent ethics committee of the Medical University of Gdansk (approval NKEBN/376/2014), and all the participants provided written informed consent.

**Figure 1:**
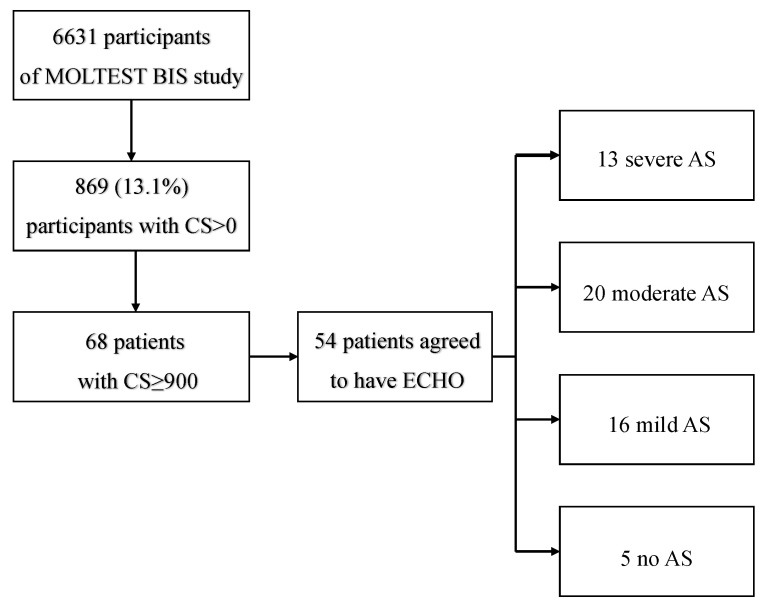
Algorithm of patient inclusion.

In the present study, all LDCTs were assessed for aortic valve calcification with the use of the CS. Patients with a CS ≥ 900, as previously described [12], were referred for ECHO examination. The patient inclusion algorithm is presented in Figure 1. Patients with clinically significant AS were also evaluated for coronary artery calcifications (CAC) score based on the Agatston method, which was modified due to the non-ECG-gated acquisition and total CS [13,14]. CAC score was stratified as follows:

0. CS—no identifiable atherosclerotic plaque on LDCT
I. 1–10 CS—minimal plaque burden on LDCT
II. 11–100 CS—definite, at least mild plaque burden on LDCT
III. 101–400 CS—definite, at least moderate plaque burden on LDCT
IV. Over 400—extensive plaque burden on LDCT [15].

LDCT scans were performed in the Radiology Department at the Medical University of Gdansk using a 64-slice CT scanner (Lightspeed VCT, GE Healthcare, Milwaukee, WI, USA) without intravenous contrast agent administration. The scanning parameters were as follows: 120 kV tube voltage, 20–30 mAs tube current, and 1.25 mm slice thickness at the mediastinal window. CTs were not electrocardiographic (ECG) gated.

The quantitative evaluation of calcifications was performed with the application of the Agatston method, which was modified due to the non-ECG-gated acquisition [13]. CS was calculated automatically during the scan analysis.

ECHO examination was performed in the group of participants with a CS of at least 900 at the aortic valve. Standard ECHO parameters were assessed, diagnosis of AS was confirmed, and AS severity was classified as either mild, moderate, or severe. Thereafter, patients were pooled twice into two groups each time: first, those with clinically significant moderate and severe AS and those without AS and with non-clinically significant mild AS; second, those with severe AS versus the rest (moderate, mild, or no AS). This was done for the purpose of plotting the receiver operating characteristic (ROC) curve and assessing CS cutoff points for clinically significant AS. The score of 900 was based on the ESC’s Valvular Heart Disease Guidelines [3]. Transthoracic ECHO examination was performed at the First Department of Cardiology at the Medical University of Gdansk using a Vivid E95 echocardiograph (GE Healthcare, Norway, Horton).

## 3. Statistical Analysis

Normality of distribution was assessed visually with the use of histograms. Descriptive statistics were performed with the use of mean and standard deviations. A one-tailed ANOVA test was used to compare the average CS values between mild and moderate, mild and severe, and moderate and severe AS. ROCs were plotted, and cutoff points were selected for comparison of groups as specified above. Area under the curve (AUC), standard error (SE), and 95% confidence intervals (95% CIs) were calculated. All calculations were performed in Prism (GraphPad Software, Inc., San Diego, CA, USA).

## 4. Results

**Figure 2:**
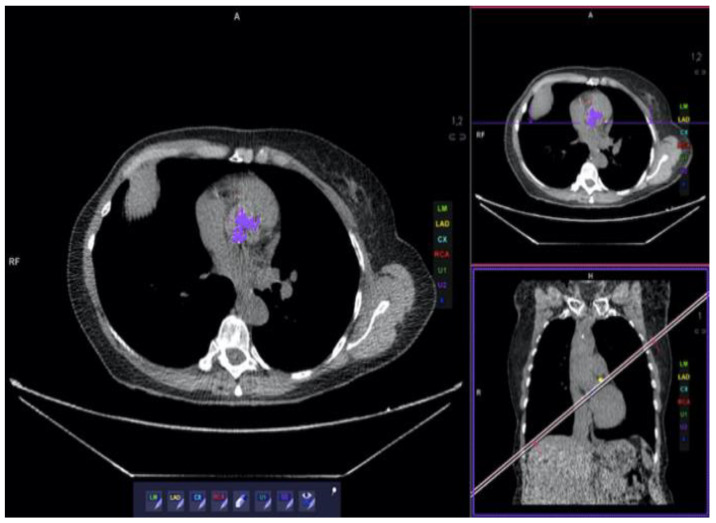
Patient with massive calcification. The calcium score was 4075. In echocardiography, severe aortic stenosis was diagnosed.

Out of 6631 MOLTEST BIS patients, 869 (13.1%) were identified as having any degree of aortic valve calcification (CS > 0) (females, *n* = 312, 35.9%). Sixty-eight (7.8%, females *n* = 17, 25%) of the 869 participants were eligible to be included in the study (CS ≥ 900), and 54 completed ECHO examinations. Based on ECHO examinations, 5 (9.26%) patients had no AS, and 49 (90.74%) had any degree of AS. Of patients with confirmed AS, 16 (29.63%) were qualified as having mild AS, 20 (37.04%) as moderate, and 13 (24.07%) as severe. Figure 2 presents a patient with massive calcification and severe AS. After pooling, as described in the methods section, 33 patients were qualified as having clinically significant AS and 21 as having clinically insignificant AS. Baseline characteristics of included patients are presented in Table 1. CS results are presented in Table 2. Ten out of 33 patients with significant AS had CAC Score 0 (30.3%), 4 had CAC Score II (12.1%), 6 had CAC Score III (18.2%), and 13 had CAC Score IV (39.4%). None of the patients had CAC Score I.

**Figure 3:**
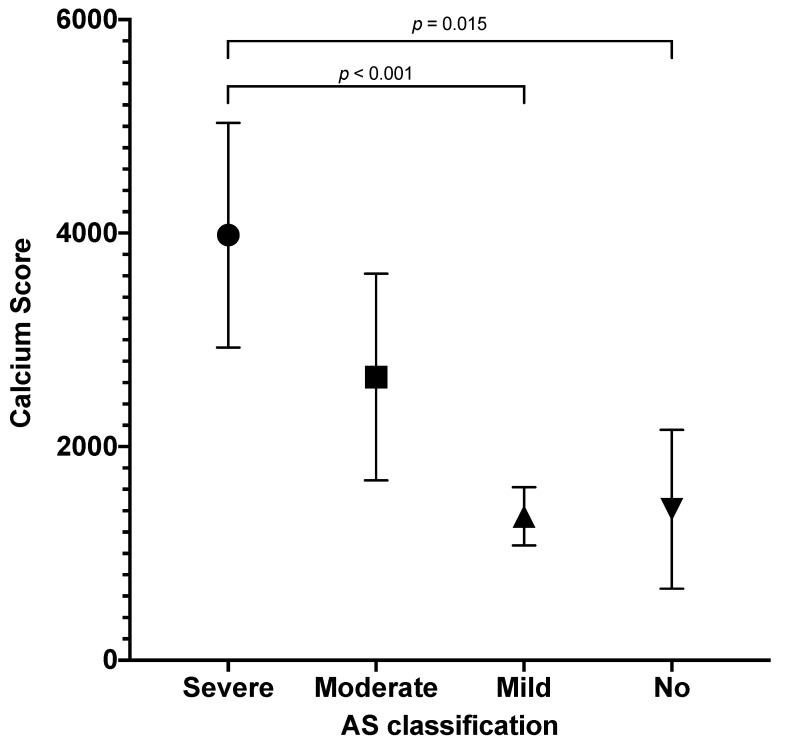
Calcium score stratified by AS category. Mean with 95% confidence intervals. Significant results of ANOVA test denoted.

Comparison of CS with the use of ANOVA revealed significant differences between AS categories (*p* < 0.001). In the sub-analysis of categories, we found significant differences between CS in severe versus mild AS (*p* < 0.001) and severe versus no AS (*p* = 0.015). Results are presented in Figure 3. Comparisons of severe versus moderate and moderate versus mild AS were not significant (*p* = 0.065, both).

**Figure 4:**
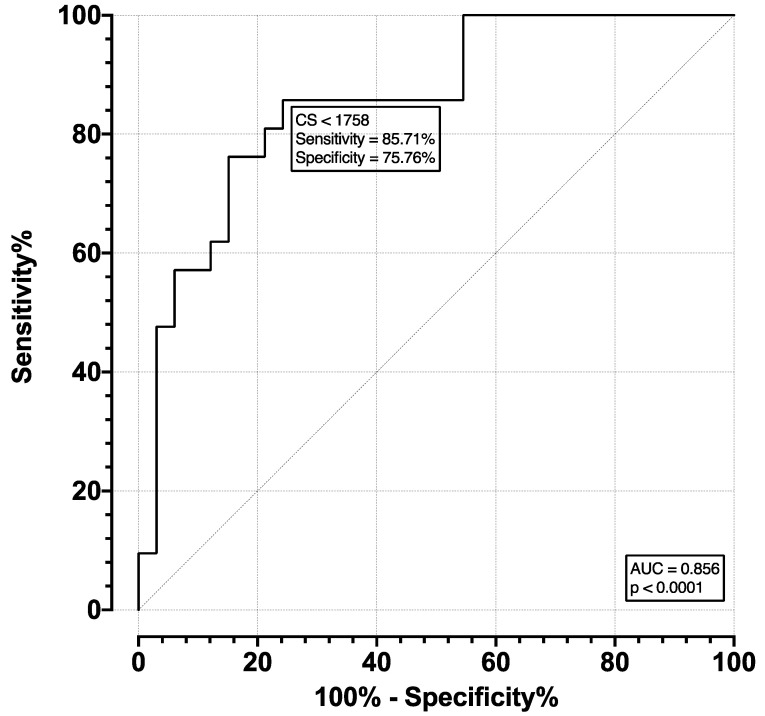
ROC for detection of at least moderate AS.

**Figure 5:**
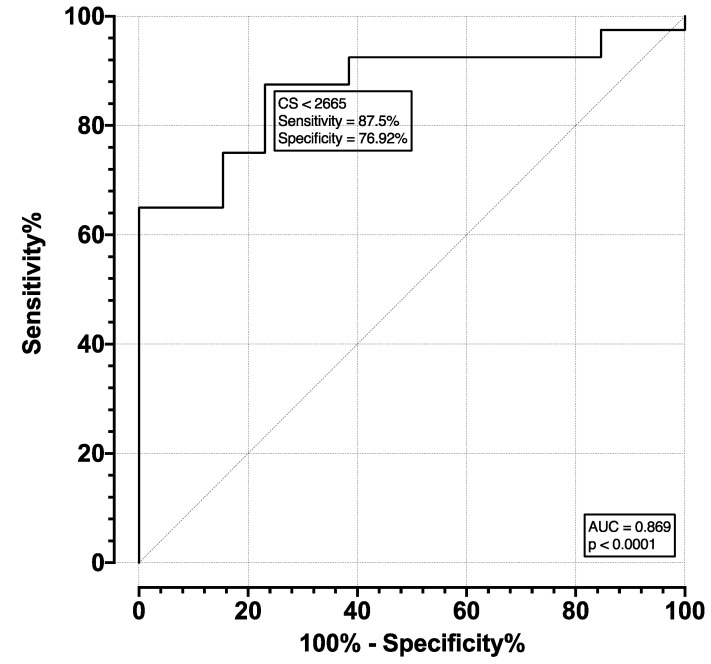
ROC for detection of severe AS.

The ROC for moderate and severe AS (pooled) versus mild and no AS (pooled) was plotted and is shown in Figure 4. The AUC was 0.856, with an SE of 0.052 and *p* < 0.001. The optimal cutoff point was selected as a CS of 1758, which corresponds to a sensitivity of 85.71% (CI 65.36–95.02%) and specificity of 75.76% (CI 58.98–87.17%). The ROC of severe versus moderate, mild, and no AS (pooled) was plotted and is shown in Figure 5. The AUC was 0.869, with an SE of 0.05 and *p* < 0.001. The optimal cutoff point was selected as a CS of 2665, which corresponds to a sensitivity of 87.5% (CI 73.89–94.54%) and specificity of 76.92% (CI 49.74–91.82%).

## 5. Discussion

To the best of our knowledge, the present study is the first to assess possible CS cutoff points for the diagnosis of clinically significant AS detected by LDCT. We performed an analysis of the CS in a substantial group of 6631 individuals undergoing LDCT for lung cancer screening, of whom 54 met the inclusion criteria and underwent ECHO examination for confirmation and assessment of AS. Based on that data, we report CS cutoff points of 1758 for diagnosing at least moderate AS and 2665 for diagnosing severe AS.

Initial studies about the usefulness of CT in the assessment of AS were published more than a decade ago. Clevel et al. established CS thresholds for each gender, above which AS can be considered probably severe (CS for women ≥ 1274; CS for men ≥ 2065) [2]. The latest 2021 ESC/EACTS guidelines for AS include CS cutoff values for severe AS at 1600 units for women and 3000 units for men assessed in standard-dose CT [3].

In 2011, Jacobs et al. conducted a study that assessed coronary calcification in LDCT scans performed in a population undergoing lung cancer screening. The results showed that the degree of coronary calcification was a strong predictive factor of cardiovascular events and thus could enable identification of patients with a high risk of coronary artery disease and selection of an appropriate preventive treatment [16]. This study was the pioneer work that showed the possibility of identifying diseases other than lung cancer with LDCT.

So far, LDCT has not been considered by the major cardiological societies as a modality that could be helpful in diagnosing AS, despite initial reports on the usefulness of LDCT [12,17].

The goal of this study was to determine a cutoff point for clinically significant AS in LDCT. Efficient screening and prompt diagnosis of AS are crucial as, although initially asymptomatic, this disease progresses quickly after symptoms occur and requires interventional treatment to prevent mortality. Unfortunately, the symptoms of AS are not specific and can also be associated with other smoking-related diseases, so the appropriate clinical moment for aortic valve replacement could be missed. Obtaining the cutoff point for clinically significant AS could enable selection of people who need to be treated.

The main strengths of our study include its screening setting. Because of this, we were able to analyze a considerable number of 6631 individuals in a cross-sectional study design. This provided a unique opportunity to analyze almost-real-world data free of the selection bias of already having a suspicion of AS. In the present study, we report an AS prevalence of approximately 1%, which corresponds to data reported in the literature for similar age groups. Furthermore, the cross-sectional design allowed for comparisons between patients with and without AS with no need for matching. This increases the potential generalizability of the reported results.

However, the present study has its limitations. First, although the initial study sample of 6631 individuals was substantial, final analyses were performed on less than 1% of this group. Naturally, this is dictated by the prevalence of AS in the population, which, dependent on the age group, can be from less than 1% to almost 10% [18]. The overall prevalence of 0.74% found in our study corresponds to data reported in the literature. Therefore, the final study group of 54 individuals, further stratified by severity, could have influenced the analyses, making them underpowered to detect significant differences. This could be especially true for comparison of the CS in severe versus moderate and moderate versus mild AS, which resulted in borderline significant *p* values. We assume that, if we were provided with a greater number of subjects, significant differences would have been detected. A further limitation imposed by the study sample size was the lack of possibility to stratify analyses by gender. This is of interest as previous researchers reported different CSs for males versus females (higher for males). Therefore, future analyses of a greater number of participants should include such an analysis to verify our findings in both genders separately. Another limitation could be a phenomenon known as the healthy screenee bias, which might have influenced the general characteristics of the MOLTEST BIS population [19]. In effect, we can suspect that patients with symptomatic AS could be underrepresented in our study group. This, however, should not have influenced the results of our study because analysis of AS prevalence was not one of its primary aims.

## 6. Conclusions

This study is the first to assess CS cutoff points for diagnosing clinically significant AS detected by LDCT. This finding could facilitate early detection of patients with clinically significant AS and therefore identify patients who require treatment. Furthermore, LDCT could be adopted in other diagnostic settings, as this modality, when compared with standard CT, does not require ECG gating and delivery of radiation.

## Figures and Tables

**Table 1:** Study population characteristics. CS—calcium score, AS—aortic stenosis.

	MOLTEST BIS	CS > 0	AS: Mild to Severe
Number of patients, n (%)	6631	869 (13.1)	49 (0.7)
Lung cancer cases, n (%)	154 (2.3)	30 (3.5)	1 (2.1)
Age (years), median (range)	63 (50–87)	65 (50–80)	67 (53–80)
Female patients, n (%)	2829 (46.6)	312 (35.9)	12 (25.0)
Current smokers, n (%)	4814 (72.6)	597 (68.7)	38 (77.1)
Former smokers, n (%)	1817 (27.4)	272 (31.3)	11 (22.9)
Smoking duration (years), median (range)	40 (15–70)	38 (15–70)	45 (20–70)
Number of cigarettes per day, median (range)	20 (10–100)	20 (10–60)	20 (10–40)
Pack-years, median (range)	40 (30–200)	53 (30–141)	43 (30–120)

**Table 2:** The results of CS. CS—calcium score, AS—aortic stenosis.

Severity of AS	Mean CS ± SD (Range)
Severe	3981.5 ± 1741.8 (1788–7415)
Moderate	2651.8 ± 2069.1 (985–9011)
Mild	1347.4 ± 512.1 (949–2644)
No AS	1413.6 ± 598.5 (1002–2457)

## Data Availability

The data that support the findings of this study are available from corresponding author upon reasonable request.

## References

[1] Chiles C., Duan F., Gladish G.W., Ravenel J.G., Baginski S.G., Snyder B.S., DeMello S., Desjardins S.S., Munden R.F., NLST Study Team (2015). Association of Coronary Artery Calcification and Mortality in the National Lung Screening Trial: A Comparison of Three Scoring Methods. Radiology.

[2] Clavel M.A., Pibarot P., Messika-Zeitoun D., Capoulade R., Malouf J., Aggarval S., Araoz P.A., Michelena H.I., Cueff C., Larose E. (2014). Impact of Aortic Valve Calcification, as Measured by MDCT, on Survival in Patients With Aortic Stenosis. J. Am. Coll. Cardiol..

[3] Vahanian A., Beyersdorf F., Praz F., Milojevic M., Baldus S., Bauersachs J., Capodanno D., Conradi L., De Bonis M., De Paulis R. (2021). 2021 ESC/EACTS Guidelines for the management of valvular heart disease: Developed by the Task Force for the Management of Valvular Heart Disease of the European Society of Cardiology (ESC) and the European Association for Cardio-Thoracic Surgery (EACTS). Eur. Heart J..

[4] Aberle D.R., Adams A.M., Berg C.D., Black W.C., Clapp J.D., Fagerstrom R.M., Gareen I.F., Gatsonis C., Marcus P.M., Sicks J. (2011). Reduced lung-cancer mortality with low-dose computed tomographic screening. N. Engl. J. Med..

[5] Ostrowski M., Marjanski T., Dziedzic R., Jelitto-Górska M., Dziadziuszko K., Szurowska E., Dziadziuszko R., Rzyman W. (2019). Ten years of experience in lung cancer screening in Gdansk, Poland: A comparative study of the evaluation and surgical treatment of 14,200 participants of 2 lung cancer screening programmes. Interact. Cardiovasc. Thorac. Surg..

[6] Field J.K., Duffy S.W., Baldwin D.R., Whynes D.K., Devaraj A., Brain K.E., Eisen T., Gosney J., Green B.A., Holemans J.A. (2016). UK lung cancer RCT pilot screening trial: Baseline findings from the screening arm provide evidence for the potential implementation of lung cancer screening. Thorax.

[7] Zhou Q., Fan Y., Wang Y., Qiao Y., Wang G., Huang Y., Wang X., Wu N., Zhang G., Zheng X. (2018). China National Lung Cancer Screening Guideline with Low-dose Computed Tomography. Zhongguo Fei Ai Za Zhi.

[8] Rzyman W., Szurowska E., Adamek M. (2019). Implementation of lung cancer screening at the national level: Polish example. Transl. Lung Cancer Res..

[9] Heuvelmans M.A., Vonder M., Rook M., Groen H.J.M., De Bock G.H., Xie X., Ijzerman M.J., Vliegenthart R., Oudkerk M. (2019). Screening for early lung cancer, chronic obstructive pulmonary disease, and cardiovascular disease (the big-3) using low-dose chest computed tomography. J. Thorac. Imaging.

[10] Vahanian A., Alfieri O., Andreotti F., Antunes M.J., Barón-Esquivias G., Baumgartner H., Borger M.A., Carrel T.P., De Bonis M., Evangelista A. (2012). Guidelines on the management of valvular heart disease (version 2012). Eur. Heart J..

[11] Clavel M.A., Malouf J., Messika-Zeitoun D., Araoz P.A., Michelena H.I., Enriquez-Sarano M. (2015). Aortic valve area calculation in aortic stenosis by CT and Doppler echocardiography. JACC Cardiovasc. Imaging.

[12] Klein-Awerjanow K., Rzyman W., Ostrowski M., Fijalkowska J., Szurowska E., Fijalkowski M. (2021). Aortic Stenosis as an Additional Finding in Low-dose Computed Tomography Lung Cancer Screening: A Cross-Sectional Study. Ann. Intern. Med..

[13] Agatston A.S., Janowitz W.R., Hildner F.J., Zusmer N.R., Viamonte M., Detrano R. (1990). Quantification of coronary artery calcium using ultrafast computed tomography. J. Am. Coll Cardiol..

[14] Agarwal P., Prakash M., Singhal M., Bhadada S.K., Gupta Y., Khandelwal N. (2015). To assess vascular calcification in the patients of hypoparathyroidism using multidetector computed tomography scan. Indian J. Endocrinol. Metab..

[15] Shen Y.W., Wu Y.J., Hung Y.C., Hsiao C.C., Chan S.H., Mar G.Y., Wu M.T., Wu F.Z. (2020). Natural course of coronary artery calcium progression in Asian population with an initial score of zero. BMC Cardiovasc. Discord..

[16] Jacobs P.C., Gondrie M.J., van der Graaf Y., de Koning H.J., Isgum I., van Ginneken B., Mali W.P. (2012). Coronary artery calcium can predict all-cause mortality and cardiovascular events on low-dose CT screening for lung cancer. Am. J. Roentgenol..

[17] Lee H.Y., Kim S.M., Lee K.S., Park S.W., Chung M.J., Cho H., Jung J.I., Jang H.W., Jung S.H., Goo J. (2016). Quantification of aortic valve calcification detected during lung cancer screening—CT helps stratifies subjects necessitating echocardiography for aortic stenosis diagnosis. Medicine.

[18] Eveborn G.W., Schrimer H., Heggelund G., Lunde P., Rasmussen K. (2013). The evolving epidemiology of valvular aortic stenosis. The Tromso Study. Heart.

[19] Spix C., Berthold F., Hero B., Michaelis J., Schilling F.H. (2016). Correction factors for self-selection when evaluating screening programmes. J. Med. Screen.

